# The flicker Pupil Light Response (fPLR)

**DOI:** 10.1167/tvst.8.5.29

**Published:** 2019-10-17

**Authors:** Prakash Adhikari, Beatrix Feigl, Andrew J. Zele

**Affiliations:** 1 Institute of Health and Biomedical Innovation, Queensland University of Technology (QUT), Brisbane, Australia; 2 School of Optometry and Vision Science, Queensland University of Technology (QUT), Brisbane, Australia; 3 School of Biomedical Sciences, Queensland University of Technology (QUT), Brisbane, Australia; 4 Queensland Eye Institute, Brisbane, Australia

**Keywords:** flicker pupil light response, melanopsin, rod, cone, photoreceptor, spectral sensitivity

## Abstract

**Purpose:**

The photoreceptor classes driving the flicker pupil light response (fPLR) to monochromatic sinusoidal temporal modulation are largely unknown. Here, we determine the photoreceptor inputs to the fPLR.

**Methods:**

The 0.5-Hz fPLR was measured in healthy observers using a Maxwellian view (41° diameter) pupillometer at five narrowband wavelengths (short: 409 nm; intermediate: 462, 507, 530 nm; and long: 592 nm) over ∼10 log units of irradiance spanning scotopic to photopic levels (5.6 to 15.6 log quanta·cm^−2^·s^−1^; −6.9 to 3.6 log cd·m^−2^). The relative photoreceptor contributions to the fPLR were then derived from these amplitude-irradiance functions using a criterion fPLR.

**Results:**

The fPLR amplitude is small (≤ 3.9 ± 3.1%; mean ± SEM) below 8.0 log quanta·cm^−2^·s^−1^ then increases with retinal irradiance in accordance with a Hill function that asymptotes between 13.0 to 15.0 log quanta·cm^−2^·s^−1^ (wavelength dependent). The Hill slope is steepest for the intermediate wavelengths. Further increases in irradiance (>15.0 log quanta·cm^−2^·s^−1^) produce a distinct suppression of the fPLR for the intermediate wavelengths. The fPLR phase delay shows a linear decrease with increasing irradiance. The spectral sensitivity of the fPLR is dominated by inner retinal melanopsin ganglion cell and outer retinal rod photoreceptor inputs to the afferent pupil control pathway; the relative melanopsin : rhodopsin weighting decreases with the transition from photopic to scotopic lighting.

**Conclusions:**

The fPLR can be used as a marker of melanopsin and rod interactions during the flicker stimulation and to quantify their contributions to the post-illumination pupil response (PIPR).

**Translational Relevance:**

These irradiance and wavelength responses will be useful in standardizing the measurements of the fPLR using chromatic pupillometry.

## Introduction

The pupil light response tracks sinusoidal light modulations^1^ with a low-pass temporal response and peak amplitude between approximately 0.5 and 1.0 Hz,^1^–^7^ a cut-off resolution frequency in the order of approximately 8 to 9 Hz,^3^,^5^,^8^ and a phase delay relative to the input signal that increases with increasing temporal frequency.^3^,^5^,^6^,^9^,^10^ This flicker pupil light response (fPLR) has also been termed phasic pupil light response.^5^,^6^,^10^,^11^ At low photopic irradiances (11.4 log quanta·cm^−2^·s^−1^) the peak-trough amplitudes of the fPLR are similar for long (reddish) and short (bluish) wavelength lights, whereas at high-photopic irradiances (15.2 log quanta·cm^−2^·s^−1^) the fPLR is suppressed at short wavelengths.^6^

Melanopsin ganglion cells receive extrinsic outer retinal rod and cone photoreceptor signals^12^–^15^ as well as generate intrinsic signals that are transmitted to the olivary pretectal nucleus (OPN), the relay nucleus for pupil control.^16^ The relative contributions of the outer retinal rods and cones and inner retinal melanopsin ganglion cells to the tonic pupil constriction amplitude during light stimulation with aperiodic incremental pulses of varying duration (∼1–100 s) depend on stimulus wavelength, irradiance, and duration^16^–^23^; the spectral and irradiance responses of the photoreceptor contributions to the fPLR have not been determined. The fPLR has however been studied using a method of silent substitution that independently controls the relative rod, cone, and melanopsin inputs to the afferent pupil pathway^4^,^5^,^10^,^24^–^27^; under mesopic illuminations (≤11.0 log quanta·cm^−2^·s^−1^) the rod and cone signals modulate the fPLR,^4^,^5^ whereas at moderate photopic illuminations melanopsin contributes to the afferent fPLR signal along with rods and cones.^5^,^10^,^24^

In chromatic pupillometry,^16^,^28^–^36^ a narrowband test stimulus with a wavelength near the peak sensitivity of melanopsin will also activate the rod and cone photoreceptors to different degrees, depending on the spectral, temporal, spatial, and adaptation properties of the stimulus.^37^ The aim of this study was therefore to determine the relative photoreceptor contributions to the fPLR for monochromatic test stimuli used in chromatic pupillometry. We first characterize the wavelength and irradiance response functions for the fPLR. With these functions, the stimulus conditions producing the largest fPLR amplitudes can be identified to optimize the assessment of the fPLR in eyes with and without disease. Next, we estimate the photoreceptor spectral sensitivity using a criterion pupil response as a function of stimulus wavelength.

## Methods

### Participants and Ethical Approval

All experimental protocols were approved by the Queensland University of Technology Human Research Ethics Committee (approval number: 080000546) and conducted in accordance with the tenets of the Declaration of Helsinki. Written informed consent was obtained from all participants after explaining the nature of the experiment. Three emmetropic observers, all 23-year-old males, with no ocular pathology and who were not under any medication that could affect the pupil light response took part in the study. The participants had normal visual acuity (>0.0 logMAR), trichromatic color vision (Lanthony Desaturated D-15), visual fields (Humphrey 30-2, Humphrey Field Analyzer; Carl Zeiss, Meditec, Inc., Dublin, CA), central retinal thickness and retinal nerve fiber layer thickness (optical coherence tomography, Nidek RS-3000 RetinaScan Advance; Nidek Co., Ltd., Tokyo, Japan), and no lenticular opacities (Grade 0, Lens Opacities Classification System, LOCS III; Chylack et al.^38^).

### Pupillometer

A custom-built extended Maxwellian view pupillometer was used to measure the flicker pupil light response (fPLR).^36^ The pupillometer consisted of five narrowband light-emitting diode (LED) sources imaged in the pupil plane of the left eye using two Fresnel lenses (100-mm diameter, 127- and 70-mm focal lengths; Edmund Optics, Singapore) and a 5° light shaping diffuser (Physical Optics Corp., Torrance, CA) to provide a 41° diameter light stimulus (retinal image diameter: 17.9 mm). The consensual fPLR of the unstimulated fellow right eye was recorded under infrared LED illumination (*λ*_max_ = 851 nm) with a PixeLINK camera (IEEE^−1^394, PL-B741 FireWire; 640 × 480 pixels; 60 frames/s; PIXELINK, Ottawa, ON, Canada) through a telecentric lens (2/3 in, 55 mm, and 2 × extender C-Mount; Computar, Singapore). The spatial resolution of the camera was 36.5 pixel/mm (0.03 mm/pixel) in order to ensure accurate detection of the pupil margin.^39^ The stimulus presentation, pupil recording, and analysis were performed using custom MATLAB software (version 7.12.0; MathWorks, Natick, MA). The spectral outputs of the primary lights were specified based on measurements of their spectral power distributions with a Spectroradiometer (StellarNet, Tampa, FL) and irradiance (W·cm^−2^·s^−1^ and converted to log quanta·cm^−2^·s^−1^) with a calibrated ILT1700 Research Radiometer (International Light Technologies, Inc., Peabody, MA).

### Pupillometry

For each pupil measurement, the baseline pupil diameter was measured in the dark for 10 seconds prior to the onset of the 0.5-Hz sinusoidal stimulus (6 cycles, 11.9 seconds). A 0.5-Hz stimulus frequency was chosen because low-frequency temporal modulations (≤1 Hz) produce larger peak-trough fPLR amplitudes than higher frequencies (>1 Hz).^1^–^3^,^6^ The fPLR was measured at five primary wavelengths (peak: 409, 462, 507, 530, and 592 nm) (Table 1) over 8 log units of corneal irradiance ranging from 6.9 to 15.3 log quanta·cm^−2^·s^−1^ (1-log unit steps) for 409- and 592-nm lights and over 10 log units of irradiance ranging from 5.6 to 15.6 log quanta·cm^−2^·s^−1^ (1-log unit steps) for 462-, 507-, and 531-nm lights. The peak irradiance was measured at the crest of the sinusoidal stimulus cycle; the trough of the cycle was always zero. The time-averaged irradiance (*Q*_A_) was calculated as *Q*_A_ = Q × *t*/(1 + *m* × cos *ωt*), where *Q* is the irradiance at time (*t*), *m* is the Michelson contrast and *ω* is the angular frequency at *t*.^40^ Hereafter, 409 nm will be called short wavelength; 462, 507, and 530 nm will be called intermediate wavelengths; and 592 nm will be called long wavelength. For each stimulus condition, at least three repeated measurements were recorded, resulting in 153 recordings per observer; the intra-individual coefficient of variation (CV; SD/mean) was 0.15 ± 0.03 (mean ± SEM), which is below the acceptable CV criterion (≤0.2) used in the pupil literature.^36^ To eliminate the effect of prior light exposure on the PLR, the observers were pre-adapted to the dim room illumination (0.0003 lux) at the start of each testing session for 30 minutes when testing scotopic stimulus irradiances <10 log quanta·cm^−2^·s^−1^ and for 15 minutes for irradiances ≥10 log quanta·cm^−2^·s^−1^. To control for any sequence effects, the order of wavelengths was randomized; to control for any effect of melanopsin bistability, the difference between successive stimulus wavelengths was always more than 100 nm. The interstimulus interval was always greater than 3 minutes to ensure that the post-illumination pupil response (PIPR) after light offset returned to the baseline diameter in the dark before a subsequent stimulus was presented.^36^ The fPLR was measured between 10 AM and 5 PM to limit the effect of circadian variation in melanopsin contributions to the PLR.^41^ To minimize any effect of autonomic^42^ and metabolic^43^ status on the PLR, each participant was tested at the same time of the day in different sessions. To minimize any effect of fatigue and sleepiness on the PLR,^44^–^46^ individual observers were tested for 1.5 hr/d or less; each observer was tested for approximately 25 hours in total divided into approximately 20 sessions. A single pupil recording sequence was 32 seconds or less and a break of at least 3 minutes was given after each sequence; the 3-minute break was also required to ensure the PIPR returned to baseline before the consecutive sequence.^36^ To determine the time taken by the PIPR to return to baseline and so the interstimulus interval, the PIPR was measured in only one observer (O1) for 462-nm lights. There was no fixation target; during the pupil recordings conducted in the darkened laboratory (0.0003 lux), participants were instructed to look straight forward and their gaze was continuously monitored; the gaze was within 5° of the center of the optical system for all recordings.^36^ Our pilot data indicate the average pupil diameter measured under such viewing condition was 7.35 mm compared with 7.29 mm when participants fixated a target positioned at 7 cm. This 0.06 mm difference (0.8% of baseline pupil diameter) induced by accommodation would have a negligible effect on our pupil results.

**Table 1:** Wavelength, Peak Irradiance, Luminance, and Photoreceptor Excitation (α-opic lux) of the Test Stimuli Used to Measure the fPLR

Wavelength, nm (FWHM)^b^	Peak Corneal Irradiance (minimum, maximum: log quanta·cm^−2^·s^−1^)	Luminance (minimum, maximum: log cd·m^−2^)	Photoreceptor Excitation (log *α*-opic lux)^a^				
			S Cone	Melanopsin	Rod	M Cone	L Cone
409 (14)	6.9, 14.7	−6.8, 1.1	−4.9, 2.9	−5.8, 2.0	−5.9, 1.9	−6.2, 1.6	−6.3, 1.5
462 (20)	5.7, 15.6	−6.9, 3.0	−5.7, 4.2	−5.9, 4.0	−6.1, 3.9	−6.4, 3.5	−6.7, 3.2
507 (27)	5.6, 15.3	−6.2, 3.5	−7.0, 2.7	−6.0, 3.8	−6.0, 3.7	−6.1, 3.6	−6.3, 3.4
530 (31)	5.8, 15.3	−6.0, 3.6	−7.7, 1.8	−6.0, 3.5	−5.9, 3.7	−5.9, 3.6	−6.0, 3.5
592 (14)	8.2, 15.3	−3.6, 3.5	−8.1, −1.0	−5.3, 1.8	−4.5, 2.6	−3.8, 3.4	−3.6, 3.6

a Lucas et al.^47^

b FWHM, full width at half maximum (nm).

### Flicker PLR Analysis

To account for the effect of individual differences in prereceptoral filtering of the ocular media, the corneal irradiances of the primary lights were converted to retinal irradiances using the model of van de Kraats and van Norren.^48^ Given that we used a large stimulus field (41°), macular pigment prereceptoral filtering was not taken into account because the human macula up to approximately 2-mm eccentricity is devoid of melanopsin ganglion cells^13^,^49^,^50^ and the macular pigment optical density is negligible beyond 10° eccentricity.^51^

**Figure 1:**
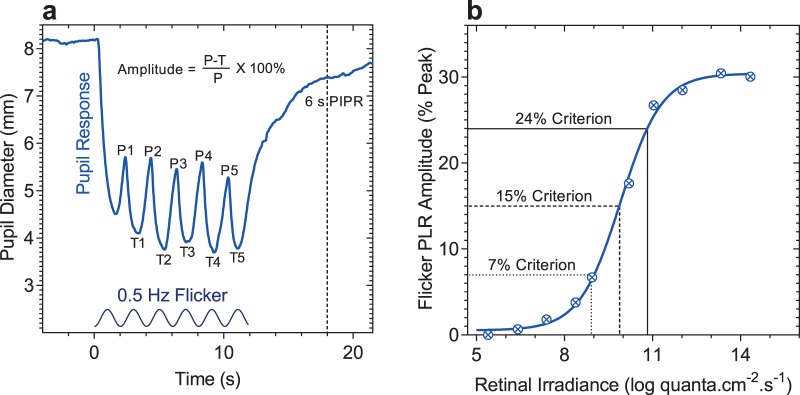
(a) Exemplar fPLR to a 0.5-Hz sinusoidal flickering light (13.6 log quanta·cm^−2^·s^−1^; 462 nm, bluish appearing); the sinusoidal stimulus is shown on the abscissa and the PIPR at 6 seconds poststimulus is indicated by the vertical dashed line. P = peak; T = trough. (b) Exemplar fPLR amplitudes as a function of the retinal irradiance of the 462-nm, 0.5-Hz flickering stimulus, the best-fitting Hill function (solid line), and three fPLR amplitudes (24% criterion, solid lines; 15%, dashed lines; 7%, dotted lines) used to determine the irradiance for the criterion amplitude for estimating the spectral sensitivity of the fPLR.

The fPLR amplitudes were defined as a percentage of the peak amplitude (Fig. 1a). The 11.9-second duration, 0.5-Hz stimuli produced a corresponding fPLR with six troughs and six peaks (6 cycles). The first trough (pupil constriction) was discarded because it does not reach its maximum amplitude due to the pupil redilation to the next stimulus cycle; the subsequent peak (pupil dilation) was therefore called P1 and the following trough was called T1; five peaks and troughs (up to P5 and T5) were considered for analysis. The fPLR amplitude was calculated by normalizing the difference between the peak and trough to the respective peak. The inter-amplitude coefficient of variation between the five peak-trough amplitudes was 0.13 ± 0.01 (mean ± SEM) on average (ranged from 0.11–0.19 for different wavelengths). Because all five peak-trough fPLR amplitudes as well as the average of the first and second amplitudes and the average of the third, fourth, and fifth amplitudes showed the same trend in the amplitude versus irradiance function and the spectral sensitivity analysis, they were averaged to calculate one fPLR amplitude per pupil recording. This averaging also minimized the intra- and inter-individual variability. That the spectral sensitivity of the fPLR does not vary over time within 11.9-second long stimulation is consistent with McDougal and Gamlin's^17^ finding with incremental pulse stimuli that the relative photoreceptor weightings to the pupil constriction are independent of stimulus duration within 17.8 seconds. To derive the phase of the fPLR with respect to the flicker stimulus, the five troughs of the fPLR were identified using a peak detection algorithm in MATLAB (R2016a; MathWorks). The phase was defined as the time difference between the fPLR trough and the respective peak of the flicker stimulus.^6^ The time difference was converted to degrees and the results were expressed as the average of the phases of the five peak-trough amplitudes. The PIPR was quantified as the pupil constriction amplitude at 6 seconds poststimulus (Fig. 1a).^36^

The fPLR data were analyzed using GraphPad prism (GraphPad software, Inc., La Jolla, CA). The fPLR amplitude (linear units) versus retinal irradiance (log units) data were described by a Hill equation,

\newcommand{\bialpha}{\boldsymbol{\alpha}}\newcommand{\bibeta}{\boldsymbol{\beta}}\newcommand{\bigamma}{\boldsymbol{\gamma}}\newcommand{\bidelta}{\boldsymbol{\delta}}\newcommand{\bivarepsilon}{\boldsymbol{\varepsilon}}\newcommand{\bizeta}{\boldsymbol{\zeta}}\newcommand{\bieta}{\boldsymbol{\eta}}\newcommand{\bitheta}{\boldsymbol{\theta}}\newcommand{\biiota}{\boldsymbol{\iota}}\newcommand{\bikappa}{\boldsymbol{\kappa}}\newcommand{\bilambda}{\boldsymbol{\lambda}}\newcommand{\bimu}{\boldsymbol{\mu}}\newcommand{\binu}{\boldsymbol{\nu}}\newcommand{\bixi}{\boldsymbol{\xi}}\newcommand{\biomicron}{\boldsymbol{\micron}}\newcommand{\bipi}{\boldsymbol{\pi}}\newcommand{\birho}{\boldsymbol{\rho}}\newcommand{\bisigma}{\boldsymbol{\sigma}}\newcommand{\bitau}{\boldsymbol{\tau}}\newcommand{\biupsilon}{\boldsymbol{\upsilon}}\newcommand{\biphi}{\boldsymbol{\phi}}\newcommand{\bichi}{\boldsymbol{\chi}}\newcommand{\bipsi}{\boldsymbol{\psi}}\newcommand{\biomega}{\boldsymbol{\omega}}\begin{equation}\tag{1}{\rm{fPLR}} = E_0 + {E_{\rm{max}} - E_0 \over 1 + 10^{(\log EC_{50} - {\rm{irradiance}})\alpha }}\end{equation}

where *E*_0_ is the baseline fPLR, *E*_max_ is the maximum fPLR, *EC*_50_ is the retinal irradiance at the semisaturation fPLR, and *α* is the slope of the Hill function. The Hill equation fit was optimized by minimizing the sum-of-square differences between the data points and the model by changing the four free parameters (*E*_0_, *E*max, *EC*_50_, and *α*).^52^ A common slope (*α*) for each wavelength was derived simultaneously using a global fit across the data from all three observers; the *E*_0_, *E*_max_, and *EC*_50_ values were derived separately for each observer. The Hill equation has been used in the literature to describe pupillo-constriction-irradiance functions.^16^

### Spectral Sensitivity Analysis

To derive the spectral sensitivity of the fPLR, the retinal irradiance required at each wavelength for the criterion fPLR amplitudes (Fig. 1b) was estimated from the best-fitting Hill equation. This approach has been widely used to derive the spectral sensitivity of the pupil light response.^16^,^17^,^19^,^20^,^53^ All criterion amplitudes were set above and below the lower and higher retinal irradiances at which the fPLR asymptotes. Retinal irradiances at the criterion fPLR were normalized to the peak and described by a best-fitting spectral nomogram computed by the following equation as defined by McDougal and Gamlin,^17^ where

\newcommand{\bialpha}{\boldsymbol{\alpha}}\newcommand{\bibeta}{\boldsymbol{\beta}}\newcommand{\bigamma}{\boldsymbol{\gamma}}\newcommand{\bidelta}{\boldsymbol{\delta}}\newcommand{\bivarepsilon}{\boldsymbol{\varepsilon}}\newcommand{\bizeta}{\boldsymbol{\zeta}}\newcommand{\bieta}{\boldsymbol{\eta}}\newcommand{\bitheta}{\boldsymbol{\theta}}\newcommand{\biiota}{\boldsymbol{\iota}}\newcommand{\bikappa}{\boldsymbol{\kappa}}\newcommand{\bilambda}{\boldsymbol{\lambda}}\newcommand{\bimu}{\boldsymbol{\mu}}\newcommand{\binu}{\boldsymbol{\nu}}\newcommand{\bixi}{\boldsymbol{\xi}}\newcommand{\biomicron}{\boldsymbol{\micron}}\newcommand{\bipi}{\boldsymbol{\pi}}\newcommand{\birho}{\boldsymbol{\rho}}\newcommand{\bisigma}{\boldsymbol{\sigma}}\newcommand{\bitau}{\boldsymbol{\tau}}\newcommand{\biupsilon}{\boldsymbol{\upsilon}}\newcommand{\biphi}{\boldsymbol{\phi}}\newcommand{\bichi}{\boldsymbol{\chi}}\newcommand{\bipsi}{\boldsymbol{\psi}}\newcommand{\biomega}{\boldsymbol{\omega}}\begin{equation}\tag{2}S(\lambda ) = {\left\{ {{{\left\{ {m\left[ {{S_{{\rm{melanopsin}}}}(\lambda )} \right]} \right\}}^{k2}} + {{\left[ {{{\left( {{{\left\{ {c\left[ {\rm{V}\lambda} \right]} \right\}}^{k1}} + {{\left\{ {r\left[ {{S_{{\rm{rod}}}}\left( \lambda \right)} \right]} \right\}}^{k1}}} \right)}^{{1 \over {k1}}}}} \right]}^{k2}}} \right\}^{1/k2}}\end{equation}

and S(*λ*) is the combined spectral sensitivity, *S*_melanopsin_(*λ*) is the action spectrum of melanopsin,^16^,^36^,^53^,^54^ V*λ* is the 10° photopic spectral luminous efficiency function,^55^ *S*_rod_(*λ*) is the Commission Internationale de l'Eclairage scotopic luminosity function (V′*λ*), and their relative contributions are defined by *m* for melanopsin, *r* for rods, and *c* for cones. The combined nomogram fit to the data was optimized by adjusting *m*, *r*, and *c* to minimize the sum of squares of the differences between *S*(*λ*) and the criterion fPLR. The *k* parameter is the summation exponent in the Quick pooling model^56^,^57^; when *k* = 1 photoreceptor contributions are summed linearly,^57^ when *k* > 1 photoreceptor contributions are summed nonlinearly, and when *k* is infinity the response is completely described by the most dominant photoreceptor. In this nomogram, *k*1 represents the combination of rod and cone contributions and *k*2 represents the combination of melanopsin with rod and cone contributions. Using *k*1 = 1 and *k*2 = 10 for the steady-state PLR (maximum constriction amplitude during light stimulation, which we call maximum PLR hereafter) with continuous incremental pulses of varying duration, the spectral sensitivity was best described by a “winner-takes-all” model.^17^ Using *k1* = 1 and *k2* = 11 for the early redilation phase of the PIPR, the spectral sensitivity was described by a nonlinear combination of melanopsin and rod contributions.^58^ For the fPLR, we fixed *k1* = *k2* = 1 based on recent findings that rod and cone inputs to the fPLR are linearly summed,^5^ and melanopsin ganglion cell inputs to the fPLR are also linearly summed with rod and cone inputs.^10^

## Results

**Figure 2:**
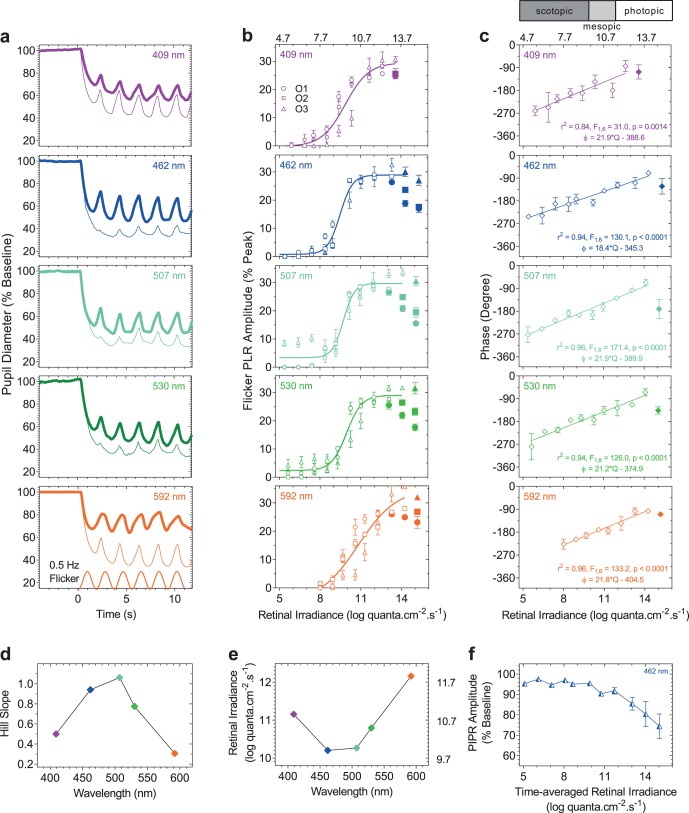
(a) Exemplar fPLR plotted as a percentage of the baseline pupil diameter (average of the 10-second prestimulus diameter in the dark) for 409-, 462-, 507-, 530-, and 592-nm narrowband lights at an average retinal irradiance of approximately 12.0 log quanta·cm^−2^·s^−1^ (thicker traces) and approximately 14.0 log quanta·cm^−2^·s^−1^ (thinner traces). At approximately 12.0 log quanta·cm^−2^·s^−1^, the fPLR amplitudes are larger with intermediate wavelengths (462, 507, 530 nm) whereas at approximately 14.0 log quanta·cm^−2^·s^−1^, the fPLR amplitudes are smaller with intermediate wavelengths due to fPLR suppression. (b) The fPLR amplitudes plotted as a function of retinal irradiance for three observers (O1, circles; O2, squares; O3, triangles) and the best-fitting Hill equations (colored lines). The suppressed amplitudes indicated by the closed symbols are not included in the global model fit. (c) The phase of the fPLR as a function of retinal irradiance (mean ± SEM; n = 3 observers) and the best-fitting linear regressions (colored lines); the phases indicated by the closed symbols are not included in the regression. ϕ = phase; Q = retinal irradiance. (d) The slopes of the Hill equations plotted as a function of stimulus wavelength. (e) Retinal irradiances required to produce the criterion fPLR (24%) as a function of stimulus wavelength. The time-averaged stimulus irradiances are given on the upper abscissa of panel b and c and the right ordinate of panel e. (f) The PIPR amplitude plotted as a function of the time-averaged retinal irradiance for 462-nm lights in Observer 1.

The peak-trough fPLR amplitude is dependent on the wavelength and irradiance of the stimulus light, with larger amplitudes for intermediate wavelengths (462, 507, and 530 nm) than the shorter (409 nm) and longer wavelengths (592 nm) as shown for an average retinal irradiance of approximately 12.0 log quanta·cm^−2^·s^−1^ (range, 11.7–12.2 log quanta·cm^−2^·s^−1^) (Fig. 2a). The relationship between the peak-trough amplitude of the fPLR and retinal irradiance for all wavelengths is well described by the Hill equation (*R*^2^ ≥ 0.88) (Fig. 2b). The fPLR amplitudes are small (≤ 3.9 ± 3.1%; mean ± SEM) at scotopic retinal irradiances <8.0 log quanta·cm^−2^·s^−1^ then increase and asymptote between 13.0 to 15.0 log quanta·cm^−2^·s^−1^. The asymptote is evident at lower irradiances for the intermediate wavelengths than with the shorter and longer wavelengths that asymptote at higher irradiances. With further increase in retinal irradiance, the fPLR amplitudes are then suppressed (Fig. 2b, closed symbols), with a larger suppression for the intermediate wavelengths compared to the shorter and longer wavelengths (Figs. 2a, 2b); note that these fPLR amplitudes that are suppressed were excluded from the Hill function fit beyond the saturation limit. The phase delay between the flicker stimulus and fPLR (Fig. 2c) decreases when increasing the retinal irradiance from 5.4 to 14.3 log quanta·cm^−2^·s^−1^ (more negative numbers indicate larger phase delays) and then increases at the highest irradiance (13.6–15.3 log quanta·cm^−2^·s^−1^) for all wavelengths. Based on evidence for a linear relationship between the light level and fPLR phase delay,^59^ a linear regression was fitted to the phase-irradiance response excluding the highest irradiance for all stimulus wavelengths. The slope of the best-fitting linear regression ranges from 18.4 to 21.9 and is not significantly different among the stimulus wavelengths (*F*_4,35_ = 0.72, *P* = 0.59).

The slope of the Hill function (Fig. 2d, Table 2) is steeper for the intermediate wavelengths (range, 0.78–1.06) compared with the shorter and longer wavelengths (range, 0.31–0.50). The retinal irradiance required to produce the same criterion peak-trough fPLR amplitude (24%) is lowest (10.2 log quanta·cm^−2^·s^−1^) for the 462-nm wavelength (Fig. 2e). The irradiances required to produce the 24% criterion fPLR range from 12.1 to 10.2 log quanta·cm^−2^·s^−1^ (i.e., photopic to mesopic); the 15% criterion range from 10.7 to 9.5 log quanta·cm^−2^·s^−1^ (mesopic to scotopic); the 7% criterion range from 9.6 to 8.9 log quanta·cm^−2^·s^−1^ (scotopic). The PIPR amplitudes (Fig. 2f) as a function of the time-averaged retinal irradiance are negligible (≲5%) at irradiances <11.72 log quanta·cm^−2^·s^−1^ and then increase with further increase in irradiance.

**Table 2:** Parameters of the Hill Function Fitted to the fPLR Amplitude-Irradiance Function (From Fig. 2a) for 409-, 462-, 507-, 530-, and 592-nm Narrowband Lights

Wavelength, nm	Hill Equation Parameters				
	*α*	*E*_0_ (% Peak)	*E*_max_ (% Peak)	Log *EC*_50_ (log quanta·cm^−2^·s^−1^)	*r*^2^
409	0.50	−0.21	29.51	9.87	0.93
462	0.94	0.77	28.90	9.49	0.97
507	1.06	3.36	29.70	9.74	0.93
530	0.78	2.36	29.02	9.98	0.94
592	0.31	−4.97	35.16	10.80	0.88

*α* (slope), *E*_0_ (baseline flicker pupil light reflex amplitude, fPLR), *E*_max_ (maximum fPLR), and *EC*_50_ (retinal irradiance at the semisaturation fPLR).

**Figure 3:**
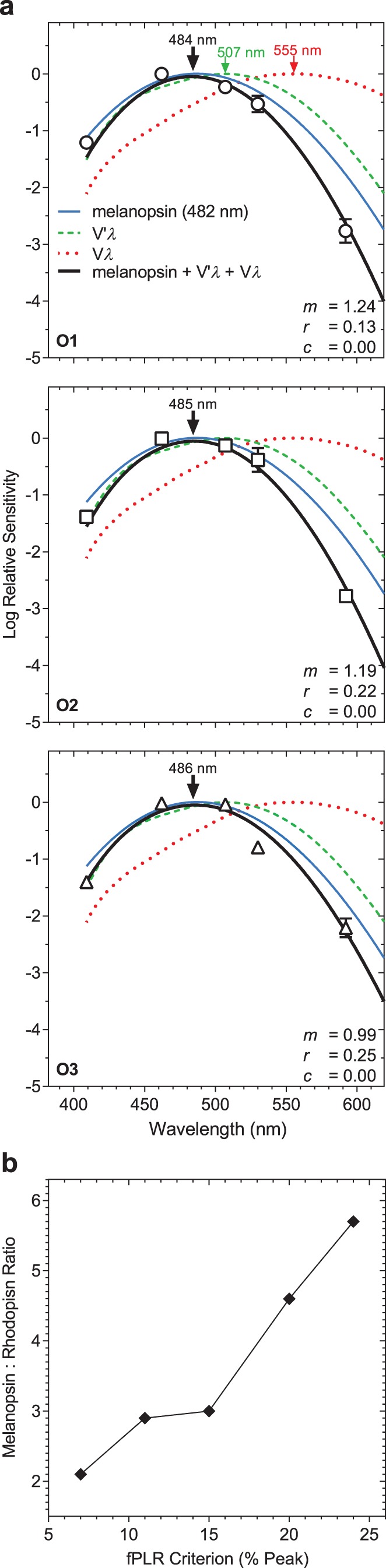
(a) Spectral sensitivity of the fPLR in the three observers (O1, O2, and O3; μ ± SEM) with a 24% criterion fPLR amplitude; m, r, and c are relative contributions to the fPLR from melanopsin, V′λ, and Vλ, respectively. The arrow indicates the peak of the nomogram. (b) Melanopsin : rhodopsin weightings ratio as a function of the fPLR criterion amplitude (7%, 11%, 15%, 20%, and 24% of the peak) derived from the global Hill function.

To determine the spectral sensitivity of the fPLR, the retinal irradiances at three criterion fPLR amplitudes (24%, 15%, and 7%) were derived from the Hill equations for each individual observer in Figure 2, then normalized, plotted as a function of wavelength (Fig. 3) and modeled with a best-fitting tertiary combination of V*λ*, V′*λ*, and melanopsin spectral nomograms (Equation 2). We set the fPLR criterion amplitudes between 7% and 24% because beyond this range, the fPLR asymptotes (Fig. 2b) and it is not possible to derive a spectral sensitivity as the fPLR amplitude would not vary with irradiance. For the 24% criterion, the best-fitting spectral nomogram peaks at 485 nm (average) and the relative photoreceptor contributions to the fPLR spectral sensitivity average 1.14 for melanopsin (*m*), 0.2 for rods (*r*), and 0.00 for cones (*c*), with melanopsin contributions 5.7× the rod contributions. For the 15% criterion, the nomogram peaks at 487 nm and the relative contributions average 0.48 for melanopsin, 0.16 for rods and 0.00 for cones, with melanopsin contributions 3.0× the rod contributions. For the 7% criterion, the nomogram peaks at 488 nm and the relative contributions average 0.21 for melanopsin, 0.10 for rods and 0.00 for cones, with melanopsin contributions 2.1× the rod contributions. The melanopsin : rhodopsin weightings ratio was determined using the global fPLR amplitude-irradiance Hill function at two additional criterion fPLR amplitudes (11% and 20%) within the lower and upper asymptotes of the function; the ratio increases almost linearly with increasing fPLR criterion amplitude (Fig. 3b). The nomogram becomes broader with lower fPLR criteria.

## Discussion

Here, we show that the fPLR measured with narrowband stimuli of different monochromatic wavelengths and irradiances produce nonselective photoreceptor activation. The spectral sensitivity derived using a best-fitting tertiary combination of the cone luminous efficiency (V*λ*), scotopic luminous efficiency (V′*λ*), and melanopsin nomogram with the assumption that their photoreceptor inputs are summated linearly^5^,^10^ shows that the fPLR is mediated by melanopsin and rhodopsin (Fig. 3). A fPLR measured with irradiances that are above and below the lower and higher retinal irradiances at which the response asymptotes (Fig. 2b) will therefore be mediated by melanopsin and rods in different relative weights. As stimulus levels decrease from photopic to scotopic, the melanopsin contribution to the fPLR also decreases relative to the rhodopsin contribution. As such, the relative photoreceptor inputs to the fPLR are irradiance dependent. The linear decrease in the fPLR phase delay with increasing irradiance suggests that the rhodopsin and melanopsin signalings may be mediated by a single pathway, potentially ipRGCs.^14^

The slope of the fPLR amplitude-irradiance Hill function (Fig. 2d) is steeper for intermediate (462, 507, and 530 nm) than shorter (409 nm) and longer (592 nm) wavelengths, consistent with the larger melanopsin and rhodopsin contributions to the fPLR.^5^ The retinal irradiance required to produce the 24% criterion fPLR amplitude is lowest for the 462-nm wavelength (Fig. 2e), which is in the vicinity of melanopsin peak sensitivity (∼480 nm) indicating that melanopsin dominates the fPLR response at high irradiances measured with narrowband lights. A recent observation that the fPLR amplitude is suppressed at high-photopic irradiances (15.2 log quanta·cm^−2^·s^−1^) compared with low-photopic irradiances (11.4 log quanta·cm^−2^·s^−1^) for shorter wavelengths (464 nm) but not for longer wavelengths (638 nm)^6^ suggests that a photoreceptor with peak sensitivity at short wavelengths is involved in this suppression. We show that this suppression occurs at higher retinal irradiances above where the fPLR amplitude asymptotes (Fig. 2b, closed symbols). There may be two explanations for this fPLR suppression. First, melanopsin cells are capable of retrograde signaling to the outer retina via dopaminergic amacrine cells^60^ to modulate the rod and cone contributions to the fPLR and suppress the peak-trough amplitude. Second, melanopsin cells can continuously signal the irradiance for at least 10 hours,^61^ and therefore can maintain a sustained pupil constriction during continuous light stimulation at high irradiances,^17^,^23^ which could lead to the lower peak-trough amplitude at the highest measured irradiances. As such, the suppression of the fPLR amplitudes is greater at the intermediate wavelengths that are closer to the peak of the melanopsin spectral response. This suppression may incur a protective function for the retina by maintaining pupil constriction to attenuate retinal illumination and limit light-induced retinal damage. Given that visual acuity is lower for bluish lights than for other wavelengths,^62^–^64^ the melanopsin-mediated fPLR suppression at high irradiances potentially optimizes visual performance at the intermediate wavelengths in the bluish-greenish region of the visible spectrum by increasing depth of focus and reducing optical aberrations.

The observed linear decrease in the phase delay (Fig. 2c) between the flicker stimulus and fPLR with increasing irradiance is consistent with Myers et al.^59^ and indicates that only one pathway may mediate the fPLR. We propose this linear fPLR phase modulation reflects ipRGC mediation of the PLR given that their selective ablation in transgenic mouse models eliminates the pupil light response.^14^ Interestingly, the fPLR phase delay increased at the highest measured irradiance for all stimulus wavelengths, which potentially corresponds to the melanopsin-mediated suppression of the fPLR amplitudes observed at these irradiances.

The irradiance required to produce the 24%, 15%, and 7% criterion fPLR ranges from photopic to scotopic irradiances (12.1–8.9 log quanta·cm^−2^·s^−1^) depending on stimulus wavelength. However, the irradiance required to produce the criterion fPLR at the wavelength of peak sensitivity ranges from 10.2 to 8.9 log quanta·cm^−2^·s^−1^; that there is very minimal or no cone activity at these irradiances around or below scotopic to mesopic transition explains the absence of cone contribution to the fPLR. There is no evidence available on how the pupil responds to flicker in coneless mice or in humans with complete cone dystrophy. In rod-/cone-less mice, at mesopic (11.3 log quanta·cm^−2^·s^−1^) as well as photopic irradiances (14.9 log quanta·cm^−2^·s^−1^), the maximum PLR to 1-Hz flicker stimuli is preserved.^65^ In humans with rod-cone dystrophy, the pupil does not track low-frequency (0.1 Hz) flicker at photopic irradiances (≥13.0 log quanta·cm^−2^·s^−1^) and instead integrates light information over time to produce the maximum PLR^4^ indicating that rods and/or cones are required to produce the fPLR. However, in line with our findings of major melanopsin contributions to the fPLR, the flicker pupil light response has also been demonstrated with melanopsin isolating stimuli (i.e., rod-/cone-silent conditions) using silent substitution techniques in humans^5^,^10^,^24^,^26^,^27^ indicating that melanopsin cells can mediate the fPLR independent of rod and cone stimulation. The change in relative melanopsin : rhodopsin input to the fPLR with lower criterion fPLR amplitudes is consistent with the studies using silent substitution.^5^,^10^ The differences in the estimated photoreceptor weights between those studies and our study are likely due to contrast differences (21%–50% in those studies^5^,^10^ versus 100% Michelson contrast in our study) that influence the effective irradiance, which also alters the photoreceptor weights. The observed melanopsin and rod dominance in the fPLR agrees with reports that rodent models with no functional rods show a significant loss of maximum pupil constriction amplitude to incremental pulses; however, those with no functional cones show normal pupil constrictions.^66^,^67^ We anticipate that the relative photoreceptor contributions to the fPLR may also change with stimulus frequency, with cone contributions possibly evident at higher temporal frequencies. The process controlling how photoreceptor inputs combine to regulate the PLR during light stimulation depends on the temporal characteristics of the stimulus, with the maximum PLR with aperiodic incremental pulses mediated via a “winner-takes-all” process^17^ but the photoreceptor inputs to the flicker PLR determined by linear summation.^5^,^10^

McDougal and Gamlin^17^ report that with aperiodic pulse stimuli, melanopsin contributions to the 1/2 maximum pupil constriction become evident only after 17.8-second duration pulses, whereas with a larger, 3/4 maximum constriction criterion, melanopsin contributions become evident with 10× shorter stimulus durations (1.78 s pulses), with melanopsin providing larger contributions than rods and cones for both conditions. Our finding that the relative melanopsin : rhodopsin input to the fPLR increases (from 2.1× to 5.7×) with increasing the criterion fPLR amplitude (from 7%–24%; equivalent to 1/4 to 3/4 maximum peak-trough fPLR amplitude) is therefore consistent with McDougal and Gamlin.^17^ Interestingly, melanopsin dominates the fPLR even for the lowest (7%) criterion (<1/2 maximum), which indicates that melanopsin may provide larger relative contributions to the fPLR with 0.5-Hz periodic flicker stimuli than to the maximum PLR to aperiodic incremental pulse stimuli. However, the PIPR amplitudes with our 462-nm, 11.9-second, 0.5-Hz flicker stimuli show a similar trend to those with 464-nm, 10-second pulse stimuli measured using the same pupillometry system and stimulus spatial configuration.^36^ The PIPR amplitudes are negligible with <11.7 log quanta·cm^−2^·s^−1^ irradiance and then increase with further increase in irradiance. Also, the 6-second PIPR amplitude with our flicker stimuli at 15.0 log quanta·cm^−2^·s^−1^ irradiance was approximately 25% of the baseline pupil diameter, which is similar to the 6-second PIPR amplitude with 10-second pulse stimuli at a comparable irradiance (14.8 log quanta·cm^−2^·s^−1^).^36^ The retinal irradiance required to produce an equivalent approximately 25% 6-second PIPR amplitude with pulse stimuli^36^ is closer to the time-averaged retinal irradiance (15.0 log quanta·cm^−2^·s^−1^) of our flicker stimuli than to the respective peak retinal irradiance (15.3 log quanta·cm^−2^·s^−1^) indicating that to produce the PIPR, melanopsin cells integrate the average irradiance information over a flicker stimulus rather than the peak irradiance; this aligns with the photon counting property of melanopsin^13^,^68^ and the temporal response of melanopsin that is independent of retinal illuminance.^26^

Our irradiance-response functions (Fig. 2) may serve as a guide to select the stimulus conditions when using monochromatic light stimuli to measure the fPLR. To apply the fPLR in clinical chromatic pupillometry studies to quantify interactions between the outer and inner retina, the level of suppression in the fPLR can be estimated as the difference in fPLR amplitudes between a long- and a short-wavelength stimulus light (i.e., the phase amplitude percentage [PAP]^11^), as demonstrated in early age-related macular degeneration^69^ and in neurologic disorders.^70^ Such retinal interactions are difficult to quantify using existing measures of retinal function, including electroretinography and automated visual perimetry. That rod contributions to the fPLR, relative to melanopsin contributions, increase with decreasing fPLR criterion amplitude which is equivalent to decreasing stimulus irradiance, the fPLR measured at irradiances less than approximately 9.6 log quanta·cm^−2^·s^−1^ will be useful in detecting rod deficits in the early stages of diseases, such as diabetes^71^,^72^ and age-related macular degeneration.^73^–^76^ With melanopsin dominating the fPLR at higher irradiances, the PAP metric can therefore be used to quantify melanopsin function at irradiance levels above the upper asymptote, and detect dysfunction when the PAP value is below normal limits. We also infer from our data that melanopsin and rod functions can be quantified by measuring the fPLR with a single monochromatic light. To maximize the dynamic range of the measurable fPLR amplitudes, irradiances near the upper asymptote should be chosen; low-frequency modulations (<1 Hz) will also extend the dynamic range because they produce larger fPLR than high frequencies (>1 Hz).^5^,^6^,^8^,^24^ A larger dynamic range of the fPLR allows for the study of a wider range of disease effects and increases the sensitivity of this response to differentiate photoreceptor function between healthy and diseased eyes. The PIPR amplitude measured after offset of the fPLR^6^ is equivalent to that with irradiance-matched pulses and so this technique offers an advantage for clinical studies for evaluating signature melanopsin and rod contributions to the pupil response at different irradiances and for detecting melanopsin deficits in retinal and neurodegenerative disorders when using the PAP metric, in addition to the early phase PIPR amplitude (<1.7 seconds after light offset) when rods combine with melanopsin,^58^ before the PIPR is then entirely driven by melanopsin.^16^,^58^
